# Polymorphisms within the canine *MLPH *gene are associated with dilute coat color in dogs

**DOI:** 10.1186/1471-2156-6-34

**Published:** 2005-06-16

**Authors:** Ute Philipp, Henning Hamann, Lars Mecklenburg, Seiji Nishino, Emmanuel Mignot, Anne-Rose Günzel-Apel, Sheila M Schmutz, Tosso Leeb

**Affiliations:** 1 Institute of Animal Breeding and Genetics, University of Veterinary Medicine Hannover, Bünteweg 17p, 30559 Hannover, Germany; 2 Department of Pathobiology, College of Veterinary Medicine, Texas A&M University, College Station, TX 77843-4467, USA; 3 Center of Narcolepsy Department of Psychiatry Stanford University School of Medicine, 701 Welch road B, Palo Alto CA 94304-5742, USA; 4 Institute for Reproductive Medicine, University of Veterinary Medicine Hannover, Bünteweg 15, 30559 Hannover, Germany; 5 Department of Animal and Poultry Science, University of Saskatchewan, Saskatoon, Saskatchewan, Canada S7N 5A8

## Abstract

**Background:**

Pinschers and other dogs with coat color dilution show a characteristic pigmentation phenotype. The fur colors are a lighter shade, e.g. silvery grey (blue) instead of black and a sandy color (Isabella fawn) instead of red or brown. In some dogs the coat color dilution is sometimes accompanied by hair loss and recurrent skin inflammation, the so called color dilution alopecia (CDA) or black hair follicular dysplasia (BHFD). In humans and mice a comparable pigmentation phenotype without any documented hair loss is caused by mutations within the melanophilin gene (*MLPH*).

**Results:**

We sequenced the canine *MLPH *gene and performed a mutation analysis of the *MLPH *exons in 6 Doberman Pinschers and 5 German Pinschers. A total of 48 sequence variations was identified within and between the breeds. Three families of dogs showed co-segregation for at least one polymorphism in an *MLPH *exon and the dilute phenotype. No single polymorphism was identified in the coding sequences or at splice sites that is likely to be causative for the dilute phenotype of all dogs examined. In 18 German Pinschers a mutation in exon 7 (R199H) was consistently associated with the dilute phenotype. However, as this mutation was present in homozygous state in four dogs of other breeds with wildtype pigmentation, it seems unlikely that this mutation is truly causative for coat color dilution. In Doberman Pinschers as well as in Large Munsterlanders with BHFD, a set of single nucleotide polymorphisms (SNPs) around exon 2 was identified that show a highly significant association to the dilute phenotype.

**Conclusion:**

This study provides evidence that coat color dilution is caused by one or more mutations within or near the *MLPH *gene in several dog breeds. The data on polymorphisms that are strongly associated with the dilute phenotype will allow the genetic testing of Pinschers to facilitate the breeding of dogs with defined coat colors and to select against Large Munsterlanders carrying BHFD.

## Background

**Figure 1:**
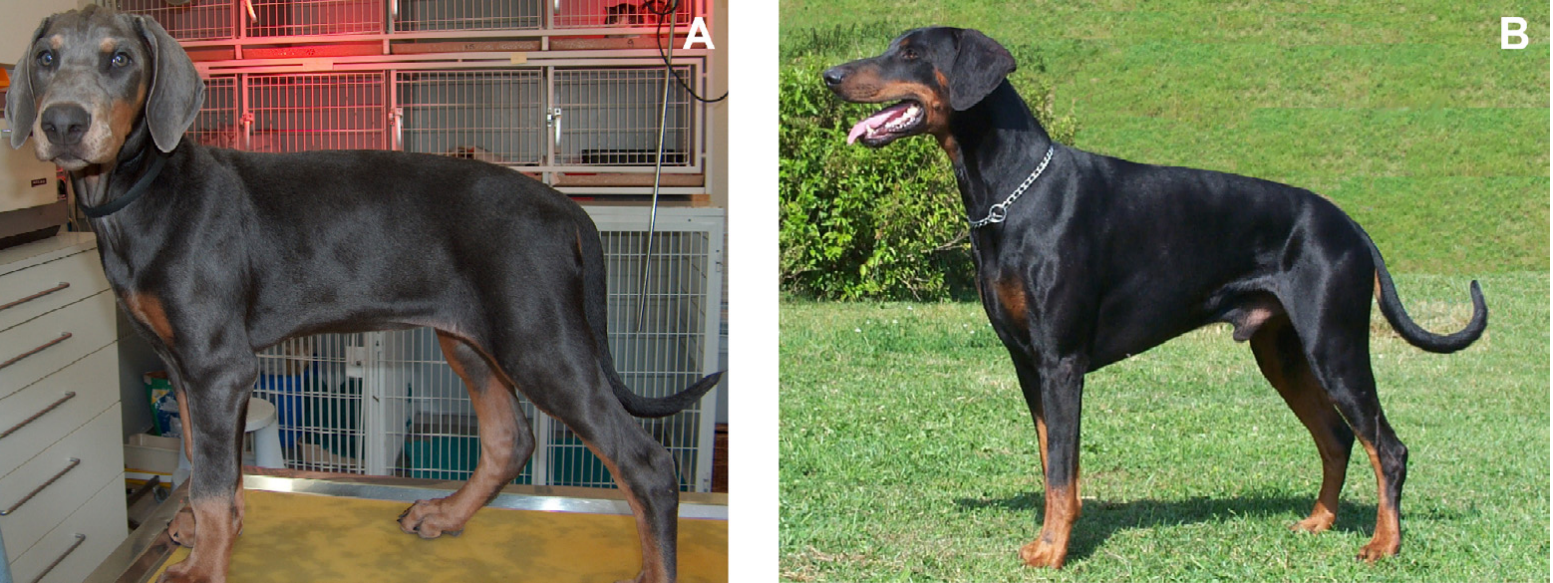
**Blue Doberman Pinscher und black-and-tan Doberman Pinscher**. Blue Doberman Pinscher (A) and black-and-tan Doberman Pinscher (B). Note the coat color differences between the two animals. The black and reddish fur parts of the black-and-tan Doberman Pinscher are changed to paler coloring in the blue dog. Classical genetics states that the blue dog is homozygous for the recessive dilute allele (d).

Coat color dilution leads to the so-called blue pigmentation phenotype in black-and-tan Pinschers (Doberman Pinschers, German Pinschers, Miniature Pinschers), characterized by a silver-blue shade of the black fur areas (Figure 1). Similarly, coat color dilution is responsible for the Isabella fawn phenotype in brown-and-tan or tan Pinschers. Color dilution in Pinschers is inherited as a Mendelian autosomal recessive trait. Although there are no severe impairments known, this pigmentation variation is of clinical relevance as Pinschers with coat color dilution show an increased prevalence of color dilution alopecia (CDA) also called Blue Doberman syndrome. CDA is characterized by a progressive loss of hair, which is sometimes accompanied by recurrent bacterial infections of the hair follicles (folliculitis). Melanosome clumping occurs within melanocytes of the epidermis and hair follicles, resulting in macromelanosomes in hair shafts that subsequently fracture when emerging from the skin. The exposed skin of CDA affected dogs is often dry and scaly as well as sensitive to sunburn or extreme cold [1]. Black hair follicular dysplasia (BHFD), a form of alopecia in various breeds where only the black coat areas are affected, is phenotypically similar to CDA [2-4].

In human and mouse, genes are already known which lead to phenotypically similar coat color variations. In mice the mutants dilute, ashen and leaden are well characterized [5-7]. These mutants correspond to the human Griscelli syndromes (GS) 1 to 3 [8-10]. Griscelli syndromes 1–3 as well as the above mentioned mouse mutants are all inherited as Mendelian autosomal recessive traits.

Mutations in three different genes, i.e. myosin Va (*MYO5A*), *RAB27A*, and melanophilin (*MLPH*), are responsible for these phenotypes. The proteins which are encoded by these genes are part of the melanosome transport complex. Therefore, in the melanocytes of affected individuals an accumulation of melanosomes around the nucleus is observed as well as large clumps of pigments in the hair shaft.

In the dilute mouse mutant the *Myo5a *gene is mutated [5], while mutations in the *Rab27A *gene lead to the ashen mouse mutant [6]. The phenotypes of these mutants are close to their human counterparts of GS1 or GS2 affected patients, respectively. Individuals carrying a mutation in one of these two genes usually develop severe neurological (GS1) or rather immunological disorders (GS2) in addition to their skin and hair color dilution [8,9]. One human case report describes that the deletion of the *MYO5A *gene exon F, which is only expressed in melanocytes, leads to hypopigmentation without further disorders [10]. In contrast to *MYO5A *and *RAB27A *mutations, which normally cause complex phenotypes, mutations in the *MLPH *gene are responsible for color dilution without any further impairment in human GS3 patients or leaden mice [7,10]. Therefore the *MLPH *gene seemed to be the most suitable candidate gene for coat color dilution in dogs and we report here the analysis of this gene in several dog breeds with an emphasis on Doberman Pinschers and German Pinschers.

## Results

### Characterization of the canine *MLPH *gene

A human *MLPH *cDNA probe was used to retrieve a canine genomic clone (RP81-203J24) from a Doberman Pinscher BAC library. A draft sequence of this 198 kb BAC clone was determined. In order to finish this draft sequence additional public whole genome shotgun sequences from a Boxer were used. The BAC clone contained the complete collagen type VI alpha 3 gene (*COL6A3*) as well as the exons 1 to 10 of the 16 exon *MLPH *gene. To obtain the missing 3'-end of the canine *MLPH *gene, Boxer whole genome shotgun sequences were assembled and joined to the sequence of the BAC clone resulting in one large contiguous sequence of 212,696 bp. Comparison of these sequences revealed a number of polymorphisms between Doberman Pinscher and Boxer DNA.

**Figure 2:**
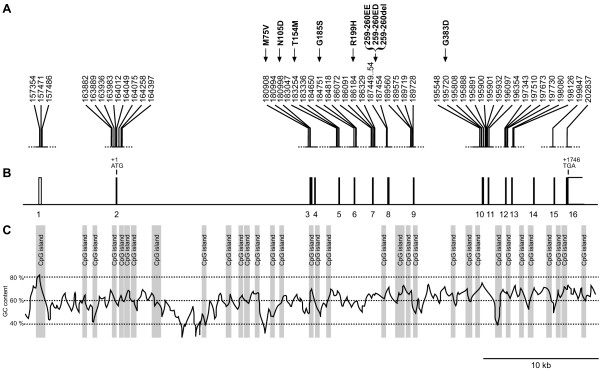
**Architecture of the canine *MLPH *gene**. (A) The 48 polymorphisms that were identified in Doberman Pinschers and/or German Pinschers are indicated. PCR products spanning each of the *MLPH *exons with adjacent flanking sequences were sequenced. The eight SNPs around exon 2 show strong association with the dilute allele in Doberman Pinschers, however they are monomorphic in German Pinschers. The R199H mutation shows strong association with the dilute allele in German Pinschers and in some Doberman Pinschers of European origin. (B) Genomic organization of the canine *MLPH *gene. Exons are denoted as boxes. Solid boxes represent coding sequence while open boxes contain the untranslated regions. The genomic section corresponds to a 50 kb interval in the analyzed sequence of 212.696 bp (positions 156.001 – 206.000). (C) Illustration of the unusually high GC-content of the canine *MLPH *gene. The GC-content was calculated using a 300 bp window. CpG island criteria were: GC > 0.5, CpG_obs_/CpG_exp _> 0.6, and length > 200 bp.

The canine *MLPH *gene spans approximately 48 kb of genomic sequence compared to 67 kb for the human *MLPH *gene. The genomic organization of the canine *MLPH *gene was inferred by comparison of the genomic dog sequence with an experimentally derived canine cDNA sequence (Figure 2). The genomic structure of the *MLPH *gene was not entirely conserved between human and dog. All but one of the 16 human *MLPH *exons could be identified in the canine *MLPH *sequence. No dog exon homologous to the human exon 9 could be identified; however, this exon is not used constitutively in all human transcripts. On the other hand, the canine *MLPH *gene contains a fifth exon of 39 bp that is not present in the human or murine *MLPH *genes. The *MLPH *gene has a very high GC-content of about 59.5%, which is significantly above the mammalian average of 41%. Consistent with the high GC-content a CpG island is located upstream of exon 1 in the dog sequence in addition to numerous CpG islands within the gene. A canonical polyadenylation signal AATAAA was identified approximately 3.1 kb downstream of the stop codon but 3'-RACE experiments indicated that in dog polyadenylation occurs only ~ 300 bp downstream of the stop codon following a sequence motif ATTGAA that weakly resembles the canonical polyadenylation signal.

The canine *MLPH *mRNA contains an open reading frame of 1746 nt encoding a protein of 581 amino acids. The canine MLPH protein was predicted to have a molecular weight of 62.7 kDa, a pI of 5.7, and shows 62% identity to the orthologous human protein (human MLPH isoform lacking the amino acids encoded by exon 9).

### Mutation analysis of the canine *MLPH *gene and association with dilute phenotype

Comparative sequencing of the exons and adjacent sequences of 6 animals from one Doberman Pinscher and 5 from one German Pinscher family revealed 43 sequence differences within these closely related breeds and an additional five variations between the breeds (Table 1). Within the Doberman Pinscher family 39 polymorphisms were observed while only 7 sequence variations were found in the German Pinscher family members. Only 3 variations segregated in both families and none of them was in the coding sequence.

**Table 1:** Polymorphisms within the canine *MLPH *gene

position^1^	cDNA position^2^	Doberman Pinscher	German Pinscher
**157354 (exon 1)**	-139 (5'-UTR)	G/T	G
**157471 (exon 1)**	-22 (5'-UTR)	A/G	A/G^3^
157486 (intron 1)		A/C^3^	A
163882 (intron 1)		C/T	T
163889 (intron 1)		A/C	C
163936 (intron 1)		A/G	A
163983 (intron 1)		A/G	A
164012 (intron 1)		A/G	A
164049 (intron 1)		A/G	A
164075 (intron 1)		C/T	C
**164258 (exon 2)**	+106	C/T (silent)	C
164397 (intron 2)		A/G	A
**180908 (exon 3)**	+223	A/G (^75^M/^75^V)	A
**180994 (exon 3)**	+309	C/T (silent)	C
**180998 (exon 3)**	+313	A/G (^105^N/^105^D)	A
183047 (intron 4)		C/G	G
**183254 (exon 5)**	+461	C/T (^154^T/^154^M)	C/T^3 ^(^154^T/^154^M)
183336 (intron 5)		C/T	C/T^3^
184650 (intron 5)		C/T	T
**184751 (exon 6)**	+553	A/G (^185^S/^185^G)	G
184818 (intron 6)		A/G	G
186072 (intron 6)		C	T
186091 (intron 6)		C	T
**186184 (exon 7)**	+596	A/G^3 ^(^199^H/^199^R)	A/G (^199^H/^199^R)
186329 (intron 7)		A	G
**187449..54 (ex. 8)^4^**	+775 – +780	indel GAGGAT +/- (indel ^259^E^260^D)	indel GAGGAG +/-^3 ^(indel ^259^E^260^E)
189560 (intron 8)		A/G	G
189575 (intron 8)		A	A/C
**189719 (exon 9)**	+1032	C/T (silent)	C
**189728 (exon 9)**	+1041	G	A/G (silent)
195548 (intron 9)		A/G	G
**195720 (exon 10)**	+1148	G (^383^G)	A (^383^D)
**195808 (exon 10)**	+1236	A (silent)	G (silent)
195888 (intron 10)		A/G	G
195891 (intron 10)		C/G	G
195900 (intron 10)		A/G	A/G
195901 (intron 10)		indel G +/-	indel G -
195932 (intron 10)		A/G	G
**196097 (exon 11)**	+1263	A/G (silent)	G
196354 (intron 11)		C/T	C
197343 (intron 11)		C/T	T
197510 (intron 11)		C/T	C
197673 (intron 12)		C/T	C
197730 (intron 12)		A/G	G
198080 (intron 12)		A	A/G
198126 (intron 12)		A/G	A/G
199847 (intron 13)		A/G	A
**202837 (exon 16)**	+1801 (3'-UTR)	C/T	C/T

^1 ^Positions refer to the entire determined genomic sequence of 212.696 bp [EMBL:BN000728].

^2 ^+1 corresponds to the adenosine of the translation start codon in the *MLPH *cDNA.

^3 ^These polymorphisms were not present in the 11 samples of the initial mutation analysis. They were identified in additionally sequenced Pinscher samples.

^4 ^Note that there are three alleles at this site (GAGGAG, GAGGAT, : : : : : :).

**Figure 3:**
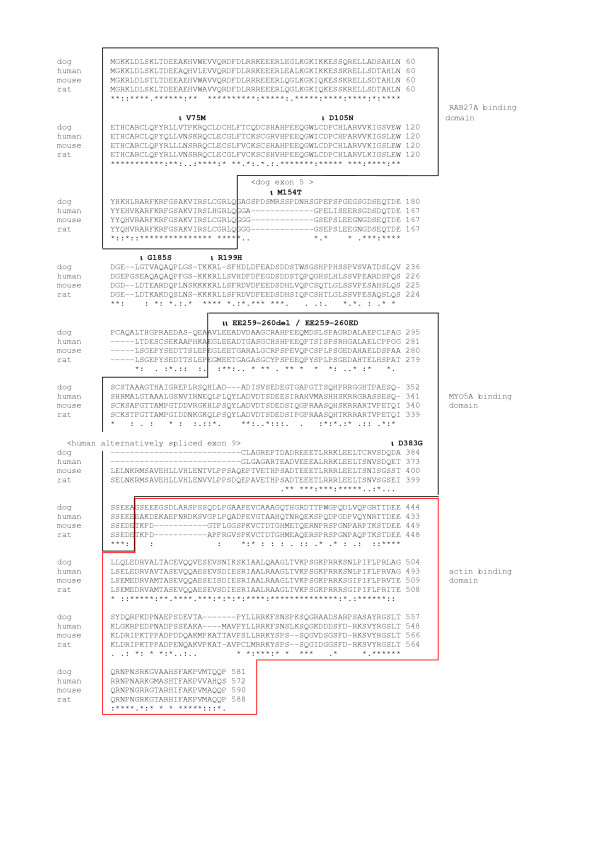
**Alignment of MLPH proteins from different species**. The MLPH protein sequences were translated from nucleotide database accessions [EMBL:AJ920333] (dog), [Genbank:AK022207] (human), [Genbank:AF384098] (mouse), and [Genbank:BC081894] (rat), respectively. The three major predicted protein domains of MLPH are indicated in accordance with [24]. Note the 13 additional amino acids in the dog MLPH protein encoded by dog exon 5, which is not conserved in other species. Another big difference between the sequences is caused by the fact that dog is lacking a homologous exon to human exon 9. In human this exon is not used constitutively and for the alignment a protein isoform without the amino acids encoded by this alternative exon was used. Polymorphisms that affect the amino acid sequence of the dog MLPH protein are indicated with arrows. None of the observed protein polymorphisms has a segregation pattern in the investigated families that would be compatible with a causative mutation for dilute.

Most polymorphisms were SNPs (46), only two indel polymorphisms were observed. Of the 48 observed polymorphisms 33 were located in introns. The remaining 15 polymorphisms were in exons, of which 7 led to amino acid exchanges in the MLPH protein (Figure 3).

**Figure 4:**
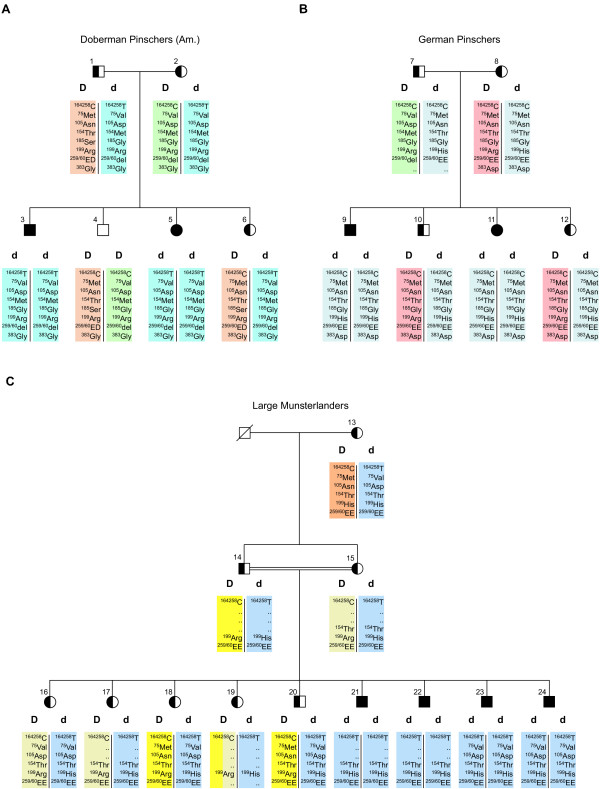
**Selected families and *MLPH *genotyping data**. (A) Doberman Pinscher family of American origin that was used in the initial mutation analysis. Dilute animals (dd) are indicated as solid black symbols. Animals 1 and 2 are obligate heterozygotes for dilute as they were black-and-tan with blue offspring. Animals 4 and 6 were classified DD and Dd based on their *MLPH *exon 2 genotypes. Genotypes for the seven polymorphic amino acid positions in the MLPH protein and the silent C/T SNP in exon 2 of the *MLPH *gene are shown. Three different marker haplotypes are color-coded. (B) Animals 8–12 of the depicted German Pinscher family were used for the initial mutation analysis. Animals 7 and 8 are obligate heterozygotes for dilute as they were black-and-tan with blue offspring. Animals 10 and 12 were classified as Dd based on their genotypes with respect to the R199H mutation. (C) Large Munsterlander family used in this study. Black symbols indicate dogs with dilute coat color and BHFD. Animals 14 and 15 are obligate heterozygotes for dilute and BHFD as they were normal with BHFD offspring. Classification of the animals 13 and 16–20, respectively, was done based on their *MLPH *exon 2 genotypes.

**Figure 5:**
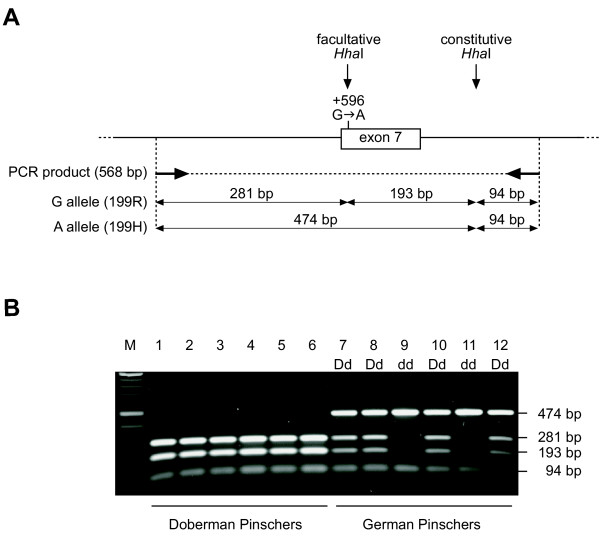
**Genotyping of the R199H mutation**. (A) Schematic diagram of the *Hha*I RFLP used for genotyping the R199H mutation. (B) Genotyping of the R199H mutation in Doberman Pinschers and German Pinschers. Numbers of the animals correspond to the numbers in Fig. 3A and 3B. Note that the Doberman Pinschers are homozygous for the presumed wildtype allele (^199^R) while in the studied German Pinscher family the R199H mutation cosegregates with the d allele.

Although an indel in exon 8 was the most striking variation observed, dogs of normal color and homozygous for each of the possible variants were encountered at this polymorphism. A G→ A transition in exon 7 causing an exchange from arginine to histidine at position 199 of the MLPH protein showed perfect co-segregation with the dilute and wildtype phenotypes in the German Pinscher family (Figure 4). We therefore established a *Hha*I RFLP assay (Figure 5) and analyzed this mutation in 341 dogs. The histidine variant was homozygous in all dilute German Pinschers (18), Beagles (2), and Large Munsterlanders with BHFD (4). Although this mutation showed strong association with the dilute phenotype in German Pinschers, it must be noted, that we observed four dogs with wildtype color from other breeds (one Large Munsterlander and three Doberman Pinschers) that were also homozygous for the 199H allele. Therefore, the R199H mutation is a tightly linked marker for the d allele in German Pinschers but it seems unlikely that it represents a loss-of-function mutation that could cause dilute coat color. The allele distribution of the R199H mutation in Doberman Pinschers turned out to be very interesting. In samples collected form Doberman Pinschers in North America the 199H allele was very rare and not obviously associated with the dilute phenotype. However, in samples from European Doberman Pinschers we observed a strong albeit not perfect association of the 199H allele with the dilute phenotype (Table 2). The available genotyping data for the amino acid changing mutations are summarized in Table 3. These data show that homozygous animals with wildtype color exist for every single amino acid replacement that we found in dilute animals.

**Table 2:** Genotype frequency of two *MLPH *polymorphisms in different breeds

Breed and phenotype	No. of animals	dilute genotype^1^	Exon 2^2^			Exon 7		
				
			CC^3^	CT	TT	^199^R^199^R	^199^R^199^H	^199^H^199^H
Doberman Pinscher (all)	140		50 (36%)	69 (49%)	21 (15%)	82 (59%)	47 (34%)	11 (8%)
wildtype coat color	98	D.	50 (51%)	48 (49%)	-	62 (63%)	33 (34%)	3 (3%)
wildtype coat color	21	Dd	-	21 (100%)	-	9 (43%)	12 (57%)	-
dilute coat color	21	dd	-	-	21 (100%)	11 (52%)	2 (10%)	8 (38%)
								
Doberman Pinscher (American origin)	38		6 (16%)	21 (55%)	11 (29%)	35 (92%)	3 (8%)	-
wildtype coat color	19	D.	6 (32%)	13 (68%)	-	16 (84%)	3 (16%)	-
wildtype coat color	8	Dd	-	8 (100%)	-	8 (100%)	-	-
dilute coat color	11	dd	-	-	11 (100%)	11 (100%)	-	-
								
Doberman Pinscher (European origin)	102		44 (43%)	48 (47%)	10 (10%)			
wildtype coat color	79	D.	44 (56%)	35 (44%)	-	46 (58%)	30 (38%)	3 (4%)
wildtype coat color	13	Dd	-	13 (100%)	-	1 (8%)	12 (92%)	-
dilute coat color	10	dd	-	-	10 (100%)	-	2 (20%)	8 (80%)
								
German Pinscher	143		143 (100%)	-	-	64 (45%)	61 (43%)	18 (13%)
wildtype coat color	117	D.	117 (100%)	-	-	64 (55%)	53 (45%)	-
wildtype coat color	8	Dd	8 (100%)	-	-	-	8 (100%)	-
dilute coat color	18	dd	18 (100%)	-	-	-	-	18 (100%)
								
Beagle	6		1	3	2	1	3	2
wildtype coat color	2	D.	1	1		1	1	
wildtype coat color	2	Dd		2			2	
dilute coat color	2	dd			2			2
								
Large Munsterlander	12		-	8	4	-	7	5
wildtype coat color	8	Dd	-	8	-	-	7	1
dilute coat color & BHFD	4	dd	-	-	4	-	-	4
								
Weimeraner (dilute coat color)	1	dd		1			1	
Am. Staffordshire (dilute col. & CDA)	1	dd		1			1	
Mountain Dogs	26	D.	25	1		25	1	
Breeds unspecified	12	D.	10	2		9	3	
								
total	341		229 (67%)	85 (25%)	27 (8%)	181 (53%)	124 (36%)	36 (11%)

^1 ^A dog with wildtype coat color has the dilute genotype DD or Dd. If the animal has never produced any dilute (dd) offspring, the genotype can not be deduced unambiguously from the phenotype and the genotype is then denoted "D.". Among the Pinschers with wildtype coat colors there were also obligate carriers of the dilute allele (Dd), as these animals had dilute (dd) progeny.

^2 ^This polymorphism corresponds to the silent C/T SNP in exon 2 at position 164258 in the reference sequence [EMBL:BN000728].

^3 ^The following associations were highly significant in Pinscher breeds under Fisher's Exact Test (p < 0.001): Exon 2 for all Doberman Pinschers, American Doberman Pinschers, and European Doberman Pinschers; exon 7 for European Doberman Pinschers and for German Pinschers. Non-Pinscher breeds were not evaluated statistically because of their small sample numbers.

**Table 3:** Genotype data of amino acid changing polymorphisms

	Large Munsterlander		Doberman Pinschers		German Pinschers	
	
Polymorphism	wild type	dilute	wild type	dilute	wild type	dilute
Exon 3 (V75M)						
VV	-	-	2	5	-	-
VM	-	-	11	-	4	-
MM	-	-	4	-	21	9
						
Exon 3 (D105N)						
DD	-	-	2	5	-	-
DN	-	-	11	-	4	-
NN	-	-	4	-	21	9
						
Exon 5 (M154T)						
MM	-	-	5	11	2	-
MT	-	-	22	2	7	-
TT	6	4	34	6	45	14
						
Exon 6 (G185S)						
GG	-	-	6	7	17	9
GS	-	-	5	-	-	-
SS	-	-	-	-	-	-
						
Exon 7 (R199H)						
RR	-	-	71	11	64	-
RH	7	-	45	2	61	-
HH	1	4	3	8	-	18
						
Exon 8 (259–260)						
del/del	-	-	1	2	-	-
del/ED	-	-	3	-	-	-
del/EE	-	-	-	-	1	-
ED/ED	-	-	-	-	-	-
ED/EE	-	-	-	-	-	-
EE/EE	7	4	-	-	3	2
						
Exon 10 (D383G)						
DD	-	-	-	-	3	2
DG	-	-	-	-	-	-
GG	-	-	4	2	-	-

A set of eight SNPs around exon 2 showed perfect association with the dilute allele in all 140 Doberman Pinschers that were analyzed. From the available genotyping data four haplotypes could be reconstructed (Table 4). A single haplotype (termed haplotype 2) was associated with the d allele in all Doberman Pinschers as well as in one Beagle family and the Large Munsterlander family segregating for BHFD. However, all German Pinschers under investigation were monomorphic around exon 2 and had the common haplotype 3. A PCR-RFLP assay was developed for the silent C/T SNP in exon 2 (position +106 in the *MLPH *cDNA sequence) since the presence of a T at this position was unique to haplotype 2.

**Table 4:** Haplotype frequencies of the eight SNPs around the *MLPH *exon 2

		Wildtype color, 305 animals		Dilute color, 45 animals	
		
Haplotype	Alleles^1^	N	%	N	%
Haplotype 1	AAAAACCG	34	5.6	-	-
Haplotype 2	AGGGGTTG	80	13.1	52^2^	57.8
Haplotype 3	CAAAACCA	493	80.8	38^3^	42.2
Haplotype 4	AAAAGCCG	3	0.5	-	-

^1 ^The alleles correspond to the eight polymorphic positions between 163889 and 164397 in the genomic reference sequence.

^2 ^Each of the 22 analyzed dilute Doberman Pinschers was homozygous for haplotype 2.

^3 ^Each of the 18 analyzed dilute German Pinschers was homozygous for haplotype 3.

While polymorphisms at the 5'-end of *MLPH *are tightly associated with dilute in American Doberman Pinschers and polymorphisms at the 3'-end are tightly associated with dilute in German Pinschers, a large group of dogs including European Doberman Pinschers, Large Munsterlanders and Beagles show strong association of dilute with markers across the entire *MLPH *gene. Detailed inspection of the available dilute chromosomes across different breeds revealed that all dilute chromosomes belonged to three different *MLPH *marker haplotypes. Each of the three families shown in Figure 4 carries one of these dilute haplotypes. A detailed comparison of the three dilute haplotypes is given in Table 5. The three different dilute haplotypes do not share extended haplotype blocks within the coding region of the *MLPH *gene. However, they do share the first three marker alleles from the region around exon 1. Thus it is possible that a single ancestral founder mutation within the promoter of the *MLPH *gene followed by subsequent recombinations is responsible for the observed diversity of dilute haplotypes.

**Table 5:** dilute haplotypes within the canine *MLPH *gene

position^1^	cDNA position^2^	Doberman Pinscher/ Large Munsterlander/ Beagle	Doberman Pinscher (American origin)	German Pinscher
**157354 (exon 1)**	-139 (5'-UTR)	G	G	G
**157471 (exon 1)**	-22 (5'-UTR)	A	A	A
157486 (intron 1)		A	A	A
163882 (intron 1)		C	C	T
163889 (intron 1)		A	A	C
163936 (intron 1)		G	G	A
163983 (intron 1)		G	G	A
164012 (intron 1)		G	G	A
164049 (intron 1)		G	G	A
164075 (intron 1)		T	T	C
**164258 (exon 2)**	+106	T	T	C
164397 (intron 2)		G	G	A
**180908 (exon 3)**	+223	G	G	A
**180994 (exon 3)**	+309	C	T	C
**180998 (exon 3)**	+313	G	G	A
183047 (intron 4)		C	G	G
**183254 (exon 5)**	+461	C	T	C
183336 (intron 5)		C	T	C
184650 (intron 5)		C	T	C
**184751 (exon 6)**	+553	G	G	G
184818 (intron 6)		G	A	G
186072 (intron 6)		T	C	T
186091 (intron 6)		T	C	T
**186184 (exon 7)**	+596	A	G	A
186329 (intron 7)		G	A	G
**187449..54 (ex. 8)^4^**	+775 – +780	GAGGAG	del	GAGGAG
189560 (intron 8)		G	G	G
189575 (intron 8)		C	A	C
**189719 (exon 9)**	+1032	C	C	C
**189728 (exon 9)**	+1041	n.d.	G	A
195548 (intron 9)		n.d.	G	G
**195720 (exon 10)**	+1148	n.d.	G	A
**195808 (exon 10)**	+1236	n.d.	A	G
195888 (intron 10)		n.d.	G	G
195891 (intron 10)		n.d.	G	G
195900 (intron 10)		n.d.	G	G
195901 (intron 10)		n.d.	G	del
195932 (intron 10)		n.d.	G	G
**196097 (exon 11)**	+1263	n.d.	G	G
196354 (intron 11)		n.d.	T	C
197343 (intron 11)		n.d.	T	T
197510 (intron 11)		n.d.	C	C
197673 (intron 12)		n.d.	C	C
197730 (intron 12)		n.d.	G	G
198080 (intron 12)		n.d.	A	n.d.
198126 (intron 12)		n.d.	A	n.d.
199847 (intron 13)		n.d.	A	A
**202837 (exon 16)**	+1801 (3'-UTR)	n.d.	T	C

^1 ^Positions refer to the entire determined genomic sequence of 212.696 bp [EMBL:BN000728].

^2 ^+1 corresponds to the adenosine of the translation start codon in the *MLPH *cDNA.

In order to rule out potential splicing aberrations we isolated skin RNA from a heterozygous Large Munsterlander (#15 in Figure 4), a dilute Beagle and a Beagle with wildtype color. We amplified the coding part of the *MLPH *cDNA by RT-PCR. Agarose gel electrophoresis gave no evidence for splicing aberrations or transcriptional silencing because the bands of the normal and dilute dogs were of the same sizes and comparable intensities. Sequencing of the RT-PCR products confirmed the *MLPH *polymorphisms previously obtained by comparative sequencing of genomic PCR products.

## Discussion

Pinschers affected by coat color dilution have a phenotype comparable to the leaden mouse mutant (*Mlph*^ln^). Therefore analyzing the canine ortholog of the *Mlph *gene causing this mutant in mice seemed a logical approach to elucidate the molecular basis for coat color dilution in dogs. The assignment of the canine *MLPH *gene to CF25q24 is in accordance with the location of the human and murine orthologous genes and with the known synteny data of the integrated canine map [11,12]. The orientation of the *MLPH *and *COL6A3 *genes to each other is also consistent with their orientation on the human map. The genomic structure of the *MLPH *gene is similar but not identical in dog, human, and mouse. Differences were observed with respect to the dog exon 5, which is lacking from other species and the human/mouse/rat exon 9 that could not be identified within the genomic dog sequence by sequence comparisons. All the experimental canine cDNA sequences obtained in this study lacked a corresponding sequence. As there are known splice variants in human lacking exon 9 (e.g. accession AK022207) it might be possible that this alternative exon is not conserved in the canine gene. An alternative explanation would be that the homology between the human and canine exon 9 is very low, so that it can not be identified by cross-species sequence comparison.

The Pinscher breeds are considered as closely related and sometimes Doberman Pinschers are still interbred with German Pinschers in order to modulate the size of the animals. In support of this, we observed 48 sequence polymorphisms within and between the two related Pinscher breeds, of which only five variations seemed to be breed specific. Taking into account the limited number of animals used in the mutation analysis it is quite likely that there are even less or no breed-specific polymorphisms at all in these breeds. Generally, German Pinscher sequences showed less variation than those of Doberman Pinschers. This had to be expected because the German Pinscher breed experienced a severe bottleneck after the second world war (7 founders in Germany, personal communication by breeders).

We identified a set of eight SNPs including a silent C to T change in exon 2, which are in linkage disequilibrium with the dilute phenotype in some breeds. In Doberman Pinschers, Large Munsterlanders, and in Beagles one haplotype co-segregated with the dilute phenotype.

The R199H mutation is in linkage disequilibrium with the dilute phenotype in German Pinschers. The R199H mutation also showed perfect association with dilute in the Beagle family and was strongly associated with the d allele in Doberman Pinschers from Europe but not from North America.

A Large Munsterlander family with pups affected with BHFD was included in this study. The phenotype of the BHFD affected animals is very similar to CDA affected Pinschers [2,3]. Histological analysis of skin biopsies of BHFD affected dogs showed the typical perinuclear clumping of melanosomes within melanocytes of the hair matrix, which is also observed in leaden mice and human GS3 patients. Since the same haplotype as in the dilute Doberman Pinschers cosegregated with BHFD in the Large Munsterlander family, this result supports the idea that CDA and BHFD are indeed the same disorder.

The data clearly imply that mutations in or near the *MLPH *gene are causing dilute coat color in dogs. The fact that the observed linkage disequilibrium between marker alleles and dilute is strongest around exon 2 in Doberman Pinschers and around exon 7 in German Pinschers suggests that there may be different mutations causing coat color dilution in dogs.

The newly identified polymorphisms in exon 2 of the *MLPH *gene should be suitable DNA markers for coat color dilution in Doberman Pinschers and for the BHFD allele in Large Munsterlanders. In German Pinschers the exon 7 polymorphisms can be used as a diagnostic test for the dilute allele. For Beagles a larger sample should be analyzed to confirm whether these polymorphisms are appropriate DNA markers for the coat color dilution in Beagles as well.

The data clearly imply that *MLPH *is the causative gene for dilute coat color in several dog breeds. However, the causal mutation has not yet been conclusively identified. The data are compatible with two alternative hypotheses: Dilute coat color could be caused by a single founder mutation in all investigated dog breeds. Under this scenario the observed haplotypes suggest a location of the causal mutation within the *MLPH *gene upstream of exon 2. The alternative hypothesis, which can not be ruled out at this time, would be that different independent *MLPH *mutations cause coat color dilution in dogs.

Although no single polymorphism affecting amino acids was associated with all dilute phenotypes, it is possible that multiple mutations in this gene or its promoter region are responsible as is true for the brown phenotype in dogs [13]. At this time a functional significance of a synonymous mutation, such as the C/T change in exon 2, can not be completely excluded as it has been reported that such synonymous polymorphisms may influence mRNA folding and stability thereby mediating functional effects. Such mutations can either act in isolation or in combination with other mutations in the same transcript [14]. All dogs with the TT genotype were of dilute coat color, even though the inverse was not true.

In order to fully explore the possibility of different functional *MLPH *mutations, mRNA from dogs of dilute phenotype of several breeds such as Great Dane, Newfoundland, Shar-Pei, Beagle, Doberman Pinscher and Large Munsterlander are being collected. If multiple mutations occurred or if the same mutation occurred at several points in time, the haplotypes would not be consistent in all individuals with dilute coat color. This trait is under selection in some breeds in which dilute coat color has minimal to no health associated problems and is under strong negative selection in other breeds such as Large Munsterlanders where the trait is typically associated with severe hair loss. This large variation in pleiotropic effects also suggests that multiple mutations may be involved.

In the mouse an independent gene termed suppressor of dilute (*Dsu*) is known that is able to suppress the effects of *Mlph *mutations. Mice carrying loss of function alleles at the *Mlph *and the *Dsu *loci have a coat color closely resembling the wildtype coat color [15]. So far, no equivalent *DSU *mutations have been reported in the dog. However, it seems possible that unrecognized *DSU *mutations might confound our analysis, which is based on the assumption of a strictly monogenic autosomal recessive inheritance of coat color dilution in dogs.

## Conclusion

We characterized the canine *MLPH *gene and identified 48 polymorphisms of this gene that occur in Doberman Pinschers and/or German Pinschers. Eight of these 48 polymorphisms located around exon 2 are in strong linkage disequilibrium with coat color dilution in Doberman Pinschers. A R199H mutation is in strong linkage disequilibrium with the dilute phenotype in German Pinschers. These results indicate that mutations in or near the *MLPH *gene are responsible for the coat color dilution in Pinschers. The reported polymorphisms will allow genetic testing of Doberman and German Pinschers to facilitate the breeding of dogs with specific coat colors.

## Methods

### Cloning and sequencing the *MLPH *gene

For the isolation of a canine BAC clone with the *MLPH *gene the Doberman Pinscher RPCI-81 BAC library [16] was screened with a ^32^P-labeled PCR fragment derived from the conserved 5'-end of the human *MLPH *cDNA. The donor animal for the RPCI-81 BAC library was a black-and-tan Doberman Pinscher named Grumpy. Grumpy was heterozygous at the dilute locus (Dd) as he had blue offspring. The probe sequence was amplified from the IMAGE cDNA clone IRAKp961H0816 provided by the Resource Center/Primary Database of the German Human Genome Project [17]. Hybridization was performed according the RPCI protocols [18]. End sequences of the positive BAC clone RP81-203J24 were determined on a LI-COR 4200L-2 automated sequencer (MWG, Biotech, Ebersberg, Germany) using the Thermo Sequenase Primer Cycle Sequencing Kit (Amersham Biosciences, Freiburg, Germany) and comparatively mapped to the human genome. Additionally, the size of the BAC clone was determined by pulsed field gel electrophoresis (PFGE).

For sequencing the BAC insert plasmid subclones were produced using the TOPO Shotgun Cloning Kit (Invitrogen, Karlsruhe, Germany). Plasmid DNA was isolated with the Montage Plasmid Miniprep_96 _Kit (Millipore, Eschborn, Germany). Sequencing was done on a MegaBACE capillary sequencing machine (Amersham Biosciences, Freiburg, Germany) using the Dyenamic™ Terminator Cycle Sequencing Kit (Amersham Biosciences, Freiburg, Germany) or on a LI-COR 4200L-2 automated sequencer using the Thermo Sequenase Primer Cycle Sequencing Kit. Shotgun sequences were collected until eight-fold coverage of the BAC clone was achieved. The sequences were assembled with Sequencher 4.2 (GeneCodes, Ann Arbor, MI, USA). Whole genome shotgun sequences from a Boxer were retrieved from the trace archive to fill gaps in the BAC clone sequence as well as for assembling the 3'-end of the canine *MLPH *gene that was not contained on the BAC clone [19]. Our experimental genomic sequences were deposited under accession [EMBL:AJ920047] in the EMBL database. The entire 212.696 bp contig consisting of an assembly from our experimental sequence reads as well as public WGS reads was also deposited in the EMBL database [EMBL:BN000728]. The exon/intron boundaries were determined by comparative alignment of the canine genomic sequence versus a canine cDNA sequence using LALIGN [20] and BLAST [21]. GC content and CpG islands were calculated with CpG plot [22]. The protein translation and calculation of protein molecular weight and pI was done with DNASTAR software (GATC, Konstanz, Germany).

### Sequencing the *MLPH *cDNA

Fresh skin biopsies (4 or 6 mm diameter ~ 30 to 60 mg) were either frozen in liquid nitrogen and stored at -80 °C or stored in RNAlater (Qiagen, Hilden, Germany) at -20°C. RNA could be isolated and yielded cDNA from both storage methods. RNA of skin was isolated using the Trizol™ reagent (Invitrogen, Karlsruhe, Germany) or the the Qiagen RNAeasy 96 Universal Tissue Kit (Qiagen, Hilden, Germany). cDNA synthesis was performed using oligo-dT and the SuperScript™III reverse transcriptase (Invitrogen, Karlsruhe, Germany) according to the manufacturer's instructions. For the subsequent PCR 2–3 μl of the cDNA were used in 50 μl reactions containing 20 pmol of each PCR primer, 200 μM dNTPs and 2.5 units of *Taq *DNA polymerase (Qiagen, Hilden, Germany). The entire coding sequence with the exception of the last two codons of the *MLPH *cDNA was amplified as four overlapping fragments. Two rounds of semi-nested PCR had to be performed in order to generate enough cDNA for DNA sequencing. The primers and conditions for the RT-PCRs are given in Table 6. The RT-PCR products were purified from agarose gels using QiaExII (Qiagen, Hilden, Germany) and directly sequenced with the Dyenamic™ Terminator Cycle Sequencing Kit and a MegaBACE capillary sequencer. The cDNA sequence of the canine *MLPH *gene was submitted to the EMBL nucleotide database [EMBL:AJ920333].

### Mutation analysis

DNA from approximately 350 dogs was available for various aspects of this study (140 Doberman Pinschers, 143 German Pinschers, 12 Large Munsterlanders, 6 Beagles, and ~50 dogs from other breeds or crossings). Doberman Pinscher and German Pinscher samples were collected from European and North American dogs. As the Doberman Pinschers showed some genetic differences with regard to their origin, these samples were divided into Doberman Pinschers of American origin (38 animals) and European origin (102 animals). The coat color of the dogs was recorded based on their pedigree certificates. In Pinschers there were four colors, black-and-tan, brown or red, blue, and Isabella fawn, respectively. Black-and-tan and brown or red were classified as wildtype colors, whereas blue and Isabella fawn were classified as dilute colors. Genomic DNA was isolated from blood using the QiaAmp 96 DNA Kit (Qiagen, Hilden, Germany) or the Nucleon BACC2 kit (Amersham Biosciences, Freiburg, Germany). Genomic DNAs of tissue samples from Doberman Pinschers were isolated using the Puregene Kit (Gentra, Minneapolis, MN, USA). All kits were used according to the manufacturers' instructions.

The exons of the canine *MLPH *were sequenced in 11 dog samples from Pinscher families with segregating coat color phenotypes. Six samples belonged to a Doberman Pinscher family (Fig. 4A). The other five samples belonged to a German Pinscher family (Fig. 4B, animals 8–12). Exons 2–15 were individually amplified with specific PCR primer pairs (Table 6). For the first exon two rounds of semi-nested PCR amplification were necessary to generate enough product for DNA sequencing. PCR was carried out in 20 μl reactions containing 10 ng genomic DNA according to the standard protocol advised by the manufacturer of the *Taq *DNA polymerase (GLtaq, Bremen, Germany). The subsequent sequencing of the PCR products was performed using the Dyenamic™ Terminator Cycle Sequencing Kit. The products were analyzed on a MegaBACE capillary sequencing machine. A set of eight SNPs around exon 2 was genotyped by DNA sequencing in most available DNA samples and statistically evaluated. The R199H mutation was either genotyped by DNA sequencing of the exon 7 PCR product or by RFLP analysis of this PCR product with *Hha*I on 1.5% agarose gels. The commercial use of *MLPH *based genotyping for diagnostic purposes in dogs is protected by the international patent EP04106291.

**Table 6:** PCR Primers used for the *MLPH *cDNA amplification and/or genomic *MLPH *mutation analysis

primer name	primer sequence	product size	T_M_	comments
*MLPH *cDNA, first round amplification				
Mlph_cDNA_Ex1_F1	CCT TCC TTC CCC TGT AGG AC	513 bp	52°C	
Mlph_cDNA_Ex4_R	GGA TCA CCT TGG CAC TCC			
Mlph_cDNA_Ex4_F1	GTG AAG ATC GGC TCG GTA G	686 bp	52°C	
Mlph_cDNA_Ex9_R	GGA TGC TGA GAG GTG GTG			
Mlph_cDNA_Ex7_F1	CTT GGA CTT TGA GGC AGA C	985 bp	52°C	
Mlph_cDNA_Ex14_R	ACT GAA CTT CCT TCT GAG G			
Mlph_cDNA_Ex10_F	AGA GAA GAG GAG ACC CTC	651 bp	52°C	
Mlph_cDNA_Ex16_R	GCT GGG TCA TCA CAG GC			
				
*MLPH *cDNA, second round amplification				
Mlph_cDNA_Ex1_F2	CTG TAG GAC CGG AGA GAG C	502 bp	52°C	
Mlph_cDNA_Ex4_R	GGA TCA CCT TGG CAC TCC			
Mlph_cDNA_Ex4_F2	AGA TCG GCT CGG TAG AGT G	679 bp	52°C	
Mlph_cDNA_Ex9_R	GGA TGC TGA GAG GTG GTG			
Mlph_cDNA_Ex7_F2	CTC TGA CGA CTC CAC TTG	967 bp	52°C	
Mlph_cDNA_Ex14_R	ACT GAA CTT CCT TCT GAG G			
Mlph_cDNA_Ex10_F	AGA GAA GAG GAG ACC CTC	651 bp	52°C	same primers as in first round amplification
Mlph_cDNA_Ex16_R	GCT GGG TCA TCA CAG GC			
				
*MLPH *genomic DNA				
P_Mlph_Ex1_for1	AGT GGC CTC AAG CCC TG	591 bp	56°C	
P_Mlph_Ex1_rev	ATG AGC TCC CTG AGA ACC			
P_Mlph_Ex1_for2	CTC CTC CCG AGG CTC CTG	563 bp	56°C	
P_Mlph_Ex1_rev	ATG AGC TCC CTG AGA ACC			
Mlph_Ex2_for	GTC ACC GAC ATT ATG TCA CAG	598 bp	58°C	
Mlph_Ex2_rev	CAG GCT GGA AGG TCA GAT C			
Mlph_Ex3&4_for	GAG ACC CAG GAT CGA GTC	725 bp	56°C	
Mlph_Ex3&4_rev	CAC TCA CAT TCC AAA GGA TC			
Mlph_Ex5_for	AGA AGT GAT GGA GGT CAC TG	471 bp	56°C	
Mlph_Ex5_rev	ACC GAA CAG GAA CAG GAG			
Mlph_Ex6_for	AG CTT GCC TGG ATG GAT G	582 bp	53°C	
Mlph_Ex6_rev	CTG CAG CCT CTG CCA AC			
Mlph_Ex7_for	GTC CTC AGC ACT TCT GAG	568 bp	53°C	
Mlph_Ex7_rev	GTG AGA AGC TTC TGG ACC			
Mlph_Ex8_for	CAG CGG GAT TTC TGA AAG C	483 bp	56°C	
Mlph_Ex8_rev	GTG TTG GAC AGT CAG AGT G			
Mlph_Ex9_for	CCC GCC TTT GCC TTA AGC	416 bp	56°C	
Mlph_Ex9_rev	GCT GCA AGG AGG AGC TTC			
Mlph_Ex10_for	CAG AGC CTG GCT CCT GAG	600 bp	56°C	
Mlph_Ex10_rev	CAC CTG GGA CAG GGA AGC			
Mlph_Ex11_for	AGG CGT CCA GAG GTT GTG	550 bp	55°C	
Mlph_Ex11_rev	GCC TGC TTC TGG AGA AGC			
Mlph_Ex12_for	CGC CGA CCA AGT CTT TGC	623 bp	55°C	
Mlph_Ex12_rev	TGG ACT TGA GGC CGT GTG			
Mlph_Ex13_for	CCT GTC TCC CCA GAT TCG	539 bp	55°C	
Mlph_Ex13_rev	GGG TTT GCC AAG GCT GAG			
Mlph_Ex14_for	CTC CTT TAT GCT CTG GCA C	591 bp	55°C	
Mlph_Ex14_rev	TTG TCA CAC GGA GAC ACA G			
Mlph_Ex15_for	CTC AGG CAG GCA GAC AAG	461 bp	55°C	
Mlph_Ex15_rev	TGC TCT GGG GTC CTA ACG			
P_MLph_Ex16_for	GCT TCA GAG CCT GAA ATT CT	469 bp	53°C	
P_Mlph_Ex16_rev	GAC AAA AGA TCA GGC TGG AG			

### Statistical methods

A set of eight SNPs around exon 2 was analyzed in 350 dogs of several breeds using Haplotyper 1.0 This software for haplotype inference uses the Bayesian algorithm [23]. Allele frequencies were tested for significant associations with the dilute phenotype using Fisher's Exact Test.

## Authors' contributions

UP performed the molecular genetic analyses and drafted the manuscript. HH performed the statistical analyses. LM provided initial ideas for candidate genes as well as dog samples. SN and EM established an experimental Doberman cross with segregating coat colors and provided samples from this colony. ARGA established an experimental Beagle cross with segregating coat colors and provided samples from these animals. SMS provided the BHFD samples from an experimental cross of Large Munsterlanders, performed some confirmative genotyping, and contributed to the writing of the manuscript. TL conceived the study, participated in the MLPH gene characterization, and finalized the manuscript.
